# Advances in the management of haemophilia: emerging treatments and their mechanisms

**DOI:** 10.1186/s12929-021-00760-4

**Published:** 2021-09-14

**Authors:** Dide Okaygoun, Danielle D. Oliveira, Sooriya Soman, Riccardo Williams

**Affiliations:** Imperial College London: Faculty of Medicine, Imperial College Road, London, SW7 2DD UK

**Keywords:** Haemophilia A, Haemophilia B, Factor replacement, Extended half-life, Emicizumab, Fitusiran, Concizumab, Inhibitor development, Gene therapy

## Abstract

Mainstay haemophilia treatment, namely intravenous factor replacement, poses several clinical challenges including frequent injections due to the short half-life of recombinant factors, intravenous administration (which is particularly challenging in those with difficult venous access), and the risk of inhibitor development. These impact negatively upon quality of life and treatment compliance, highlighting the need for improved therapies. Several novel pharmacological therapies developed for haemophilia aim to rebalance the clotting cascade and potentially circumvent the aforementioned challenges. These therapies utilise a range of different mechanisms, namely: the extension of the circulating half-life of standard recombinant factors; the mimicking of factor VIII cofactor activity; rebalancing of coagulation through targeting of natural anticoagulants such as antithrombin and tissue factor pathway inhibitor; and inducing the production of endogenous factors with gene therapy. These therapies carry the potential of revolutionising haemophilia treatment by alleviating the current challenges presented by mainstay factor replacement. This review will provide an overview of the key trial findings related to novel therapies based on the mechanisms described above.

## Background

Haemophilia is an X-linked recessive disorder that is divided into two different subtypes—haemophilia A (HA) and B (HB), which result from the deficiency or complete absence of clotting factors VIII (FVIII) and IX (FIX) respectively. Current management of HA and HB primarily includes prophylactic factor replacement, which is often commenced at a young age and requires multiple intravenous access weekly, and on-demand factor administration for acute bleeding [1].

A major issue with replacement factors is their immunogenic potential. Neutralising antibodies, known as inhibitors, can develop against the infused factor: approximately 30% of HA and 3% of HB patients develop inhibitors [2]. The development of these inhibitors can complicate the management of haemophilia patients—this can impact not only the quality of life (QoL) of these patients, but also their prognosis. If inhibitors develop, immune tolerance induction can potentially promote tolerance to exogenous FVIII or FIX, and bypassing agents (BPAs) such as recombinant factor VIIa (rFVIIa) and activated prothrombin complex concentrates (aPCC) can be used to circumvent factor use [3].

Haemophilia management significantly impacts QoL. Frequent factor administration is not only disruptive to daily life but can also lead to vein damage and scarring, potentially resulting in poor compliance [4]. Prophylaxis requires foreplaning, particularly for individuals participating in high-risk activities such as contact sports, limiting the activities in which they partake [5]. Finally, despite adequate prophylaxis, long-term sequelae such as joint arthropathy still occur, representing a large source of morbidity and reduced QoL [6].

Given its limitations, improving haemophilia management remains an important aim and has attracted a large amount of research. This review will discuss novel mechanisms of treating haemophilia, potentially circumventing the limitations of current factor replacement therapy.

Referenced publications were obtained through use of relevant search terms in PubMed, Mendeley, Scopus, and Google Scholar databases; and by identifying key publications in the reference lists of notable reviews and clinical trials.

## Extended half-life products

In recent years, recombinant FVIII (rFVIII) and recombinant FIX (rFIX) have undergone modification to create extended half-life (EHL) products, offering the promise of less frequent administration.

One approach to creating EHL products involves fusing FVIII or FIX to a protein with a long half-life [7]. Albumin and the constant region (Fc) of IgG have long plasma half-lives as they bind to the neonatal Fc receptor, which is critical for the endogenous recycling of both IgG and albumin [8–10]. Fc-fusion has been applied to both FVIII (efmoroctocog alfa) and FIX (eftrenonacog alfa), and albumin-fusion has been applied to FIX to produce albutrepenonacog alfa. These fusion proteins have all demonstrated improved terminal half-lives when compared to standard factor replacement and are licenced for clinical use [11–13]. Both Fc-fusion products have displayed excellent safety and efficacy profiles in adults and children [11, 14, 15], lower annualised bleeding rates (ABRs) when used prophylactically [11], and no inhibitor development [11–13, 16, 17]. Albutrepenonacog alfa also demonstrated good safety and efficacy [13, 18, 19].

Another method is PEGylation, where one or more PEG chains are covalently linked to rFVIII or rFIX. PEG chains interfere with the recombinant factors binding to their clearance receptors, thereby prolonging circulating half-life [7]. Several PEGylated rFVIII products, such as rurioctocog alfa pegol, are currently in clinical use. Rurioctocog alfa pegol manifested a 1.4- to 1.6-fold prolongation in half-life when compared to standard rFVIII [20–25]. PEGylated rFIX, nonacog beta pegol, is also in clinical use, with a fivefold increase in half-life over standard rFIX [26], as well as being well tolerated with mostly mild/moderate adverse events (AEs), no inhibitor development, and a reduction in ABRs [27]. The use of PEGylated factors in children under 12 is an important consideration with regards to PEGylated products. Turoctocog alfa pegol is licenced for use in all age groups (Food and Drug Administration (FDA) and European Medicines Agency), while the use of damoctocog alfa has been explored in the PROTECT VIII Kids trial, which found damoctocog alfa pegol to be efficacious in children with haemophilia A [28]. However, damoctocog alfa pegol and other PEGylated EHL products (namely: rurioctocog alfa pegol, nonacog beta pegol) generally are not licenced for use in children under 12 due to uncertainty regarding the long-term safety of PEGylated products in this age group.

Finally, single chain technology can be used to increase the stability of rFVIII, as well as its affinity for von Willebrand Factor (VWF), thereby decreasing its clearance and prolonging its circulating half-life [29]. Lonoctocog alfa is a novel B-domain truncated single chain rFVIII approved by the FDA, comprising covalently-linked heavy and light rFVIII chains [30]. Lonoctocog alfa exhibited an improved half-life compared with standard rFVIII, however this improvement was only slight at 14.5 h compared with 13.3 h for standard factor, meaning lonoctocog alfa requires more frequent administration when compared with other EHLs [31]. Lonoctocog alfa also demonstrated excellent safety and efficacy in both adults and children with severe HA [32, 33].

Despite the impressive results seen thus far, EHL products are not without limitations. One drawback of EHLs is that they still rely upon intravenous administration and thus still incur the complications related to this route. However, dalcinonacog alfa, a modified rFIX product, is currently in clinical trials and has been found to be amenable to subcutaneous administration. Dalcinonacog alfa is a modified FIX protein that has three amino acid substitutions incorporated, which work to increase FIX’s catalytic activity; its affinity for FVIIIa; and its resistance to inhibition via antithrombin [34]. An initial phase I/IIa trial was embarked upon which found dalcinonacog alfa to have high subcutaneous bioavailability of 8.2–20.3% and a half-life ranging from 53.9 h up to 106.9 h [35]. The amenability of dalcinonacog alfa to subcutaneous administration offers a potential improvement over both standard factor replacement and currently licenced EHLs. Furthermore, despite only being investigated with daily administration to date, dalcinonacog alfa has demonstrated itself to deliver effective steady state levels of FIX, which may owe it to less frequent dosing regimens, as seen with the other EHLs. A phase IIb study is currently in progress which will provide further insights into the pharmacokinetics, safety and efficacy of once daily subcutaneous dalcinonacog alfa [36].

The improvement in half-life of EHL rFIX products is greater than that of EHL rFVIII products. This is because the half-life of rFVIII is limited by the half-life of VWF, as the modified FVIII retains the ability to interact with VWF [37]. This less impressive improvement in half-life seen in rFVIII products could be overcome through targeting of VWF, potentially through prolongation of the life cycle of VWF or limiting VWF-mediated clearance of FVIII. This could result in greater increases in the half-life of FVIII EHL products, however this approach is not without issue. Individuals with HA have normal levels of circulating VWF, thus prolonging VWF half-life to also increase FVIII half-life could raise the level of circulating VWF in these individuals, and therefore increase the risk of arterial diseases such as heart attack or stroke.

EHL products have shown great promise as an alternative to standard factor replacement, reducing the administration frequency and thus improving patient QoL and prognosis. However, there is a lack of comparison between the different EHL products. All EHL products have been compared with their comparative standard factors, however, differing dosing, administration frequencies and patient selection processes make drawing conclusions as to their comparative efficacies difficult. Future work should include comparing the different EHL products to optimise treatment of HA and HB. Below is a table summarising all currently available EHL products (Table 1).

**Table 1:** A table summarising the currently available extended half-life products, including their mechanism of half-life prolongation, terminal half-life, ratio of terminal half-life compared with standard factor, and their licensing status in the United States and European Union

EHL product	Generic name	Mechanism of action	Half-life of EHL product (hours)	Half-life of standard factor (hours)	Ratio (EHL to standard factor half-life)	Licensing status	Frequency of administration	Comments and notable points
*FVIII extended half-life products for use in Haemophilia A*								
Lonoctocog alfa pegol	Afstyla	B-domain truncated single chain	14.5	13.3	1.1	Licensed	Every second day	Only modest improvement in half-life thus requiring more frequent administration than other EHLs
Efmoroctocog alfa	Elocta	FVIII Fc fusion protein	19.0	12.4	1.5	Licensed	Effective with once-weekly administration	
Damoctocog alfa pegol	Jivi	PEGylated B-domain deleted FVIII	18.5	13.0	1.4	Licensed	Once weekly administration possible	Not licensed for use in children under 12
Rurioctocog alfa pegol	Adynovi	PEGylated FVIII	14.3	10.4	1.4	Licensed	Twice weekly or less	
Turoctocog alfa pegol	NovoEight	PEGylated B-domain truncated FVIII	19.5	13.0	1.5	Licensed	Every 4–7 days	Can be used in all age groups, including under 12 years
*FIX extended half-life products for use in Haemophilia B*								
Nonacog beta pegol	Refixia	PEGylated B-domain truncated FIX	92.7	17.8	5.2	Licensed	Currently licenced for administration every 3–4 days	Not licensed for use in children under 12
Albutrepenonacog alfa	Idelvion	FIX albumin fusion protein	92.0	17.0	5.4	Licensed	Administration up to once every 14 days (up to 7 days in children)	
Eftrenonacog alfa	Alprolix	FIX Fc fusion protein	82.1	33.8	2.4	Licensed	Once every 7–10 days in all age groups	
Dalcinonacog alfa	N/A	rFIX with 3 amino acid mutations	106.9	21.0	5.1	Phase IIb	Currently once daily	The only EHL currently to offer subcutaneous administration

Information regarding their administration frequencies along with any notable points regarding individual EHLs are also provided

*EHL* extended half-life; *FVIII* factor VIII; *FIX* factor IX

## Emicizumab

Emicizumab, a recombinant humanised bispecific IgG antibody, mimics the cofactor function of the missing FVIII in HA. It simultaneously binds activated FIX (FIXa) and factor X (FX), bringing them into spatial proximity to promote FIXa-catalysed FX activation, thereby restoring haemostasis [38]. Emicizumab is being clinically used in HA patients with and without inhibitors.

The unique molecular structure of emicizumab means it is not subject to the same physiologic mechanisms that eliminate FVIII from circulation, hence it persists in circulation for much longer compared to FVIII. It has a long half-life of around 4–5 weeks, which supports dosing regimens once weekly, every 2 weeks or even monthly. The lack of molecular resemblance between emicizumab and FVIII also allows its use in patients with FVIII-inhibitors, making it a useful therapeutic tool where immune tolerance induction and BPA prophylaxis have failed. Further coupled by its subcutaneous route of administration, emicizumab can reduce treatment burden associated with frequent intravenous factor infusions.

The probability of developing anti-drug antibodies (ADAs) against emicizumab is low, as humanised antibodies have low immunogenicity [39]. Should ADAs develop, they are unlikely to cross-react with FVIII and compromise replacement therapy, since emicizumab and FVIII have different molecular structures [39]. This further allows the use of emicizumab in patients without FVIII-inhibitors, as well as with inhibitors.

Phase III HAVEN trials of emicizumab prophylaxis demonstrated clinically meaningful efficacy and safety across all age groups, including children [40–43]. It resulted in significantly lower ABRs compared to: no prophylaxis [40, 41]; previous BPA prophylaxis in patients with FVIII-inhibitors [40]; and previous FVIII prophylaxis in patients without FVIII-inhibitors [41]. Pharmacokinetic profile was uniform: half-life of 4–5 weeks; and sustained stable peak/trough plasma concentration at repeated injections. The latter may further reduce bleeding rates compared to treatments with more fluctuating pharmacokinetic profiles [40–42, 44].

The majority of patients did not develop ADAs [40–43], however, four patients in the HAVEN 2 trial did [43]; two had ADAs with neutralising potential associated with declining plasma emicizumab concentrations. One patient had loss of emicizumab efficacy, the other became ADA-negative after 48 weeks of detection, with restoration of emicizumab efficacy. Emicizumab efficacy was unaffected by non-neutralising ADAs [43, 44]. However, the probability of developing ADAs after long-term exposure to emicizumab and the long-term effect of ADAs on emicizumab efficacy cannot be determined as trial follow-up times do not allow for such conclusions to be drawn. Moreover, ADAs can follow different trajectories between individuals, rendering emicizumab completely ineffective, or disappearing over time [43].

The most frequent AE associated with emicizumab was injection-site reactions [40–44]. No thromboembolic events occurred, except when used in combination with aPCC for breakthrough bleeding [40–44]. aPCC supplies emicizumab with the coagulation factors needed to form the tenase complex, leading to synergistic thrombin generation, increasing the risk of thromboembolism [40]. Therefore, use of aPCC for treating breakthrough bleeding is not recommended during emicizumab prophylaxis [45].

One of the many advantages of emicizumab is its cost effectiveness. In a multicentre observational study, the average total cost of haemostatic treatment per patient (both prophylactically and for treatment of bleeds) over a 6-month period for all patients was shown to significantly decrease from $260,168 to $179,636 after initiating emicizumab when compared to patients’ prior prophylactic regimens. This cost reduction was most significant in patients with inhibitors [46].

Emicizumab is practical to use, requiring no routine laboratory monitoring [47]. However, emicizumab interferes with clot-based assays, rendering the measurement of activated partial thromboplastin time (APTT), FVIII-activity and FVIII-inhibitor titre unreliable [47]. This impedes precise laboratory monitoring of HA patients which is important when undergoing surgery or following a serious traumatic bleed, as adjunctive haemostatic treatments are likely to be needed. Chromogenic assays can be used instead to measure the aforementioned clotting parameters; however, these are unavailable in most laboratories [47]. The in vitro use of anti-emicizumab antibodies was proposed for accurate measurement of clotting parameters, which can eliminate emicizumab’s effects [48]. This requires further investigation before implementation into routine laboratory practice.

Another unresolved issue is the uncertainty surrounding perioperative management of haemostasis in patients taking emicizumab. Despite its efficacy in preventing bleeds, additional haemostatic replacement therapy is likely to be required at time of major surgery [45]. Case reports have described the successful additional use of rFVIIa in patients with FVIII-inhibitors and FVIII in patients without inhibitors, in the context of major orthopaedic surgery [45, 49, 50]. Data from surgical experience in the HAVEN 1–2 trials suggested that additional BPA administration perioperatively may not always be required, and that post-operative bleeding requiring BPA administration is unlikely [51]. This was further supported by real-world outcome data from prospective paediatric cohorts, where the majority of surgical procedures were uncomplicated and only one or two additional doses of BPAs were used [52–54]. Notwithstanding, most of the procedures described were minor, and included central venous access device removals and tooth extractions. Therefore, further studies are warranted into the efficacy of emicizumab perioperatively, in the context of both minor and major surgeries. The current recommendation provided by the United Kingdom Haemophilia Centre Doctors’ Organisation is to delay non-urgent major surgery in patients on emicizumab prophylaxis [52–54]. However, such delay may not always be possible, for instance, with unplanned emergency surgeries. In these circumstances, the lack of specific protocols and uncertainty may put patients at risk, hence, specific guidance on this matter is needed.

## Fitusiran

**Fig. 1:**
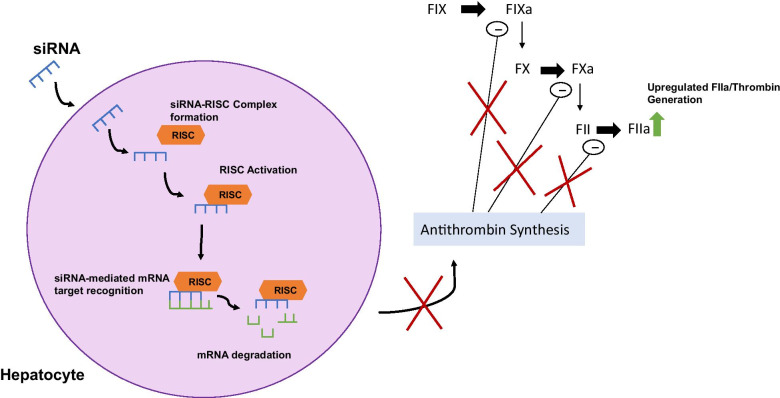
Diagram illustrating the mechanism of action of Fitusiran/ALN-AT3. GalNAc-siRNA conjugate enters hepatocytes and binds with a ribonucleoprotein to form the RNA-induced silencing complex (RISC) [56]. Once formed, it targets and degrades the SERPINC1 mRNA which encodes antithrombin, effectively silencing the gene. The consequent inhibition of antithrombin production inhibits its down-regulatory effects on FIXa, FXa and thrombin (FIIa). Subsequent increased FIIa generation will alleviate the bleeding phenotype in haemophilia A and haemophilia B. *siRNA* small interfering ribonucleic acid; *RISC* RNA-induced silencing complex; *mRNA* messenger ribonucleic acid; *FIX* factor IX; *FX* factor X; *FII* factor II/thrombin; *FIXa* activated factor FIX; *FXa* activated factor X; *FIIa* activated factor

Fitusiran (ALN-AT3), a novel therapy applicable to both HA and HB [55, 56], consists of the amino acid, N-Acetylgalactosamine (GalNAc), the ligand of the hepatic asialoglycoprotein receptors, conjugated to a synthetic siRNA [55, 56]. Administered subcutaneously, it targets and degrades a region of the SERPINC1 gene mRNA, preventing antithrombin production and enhancing thrombin generation [55] (Fig. 1). Antithrombin is a potent anticoagulant which inactivates FIXa, activated factor X (FXa) and activated factor II (FIIa/thrombin) [55]. Therefore, fitusiran can correct the coagulation imbalance and prevent the bleeding phenotype [55, 56].

In a phase I trial [57, 58] involving HA and HB patients lacking inhibitors, fitusiran increased thrombin generation in both haemophilia types, reduced ABRs, and caused dose-dependent antithrombin lowering, notably 87% at 80 mg. Pharmacokinetic analysis revealed a half-life of 2.6–5.3 h and no drug accumulation following repeated administration [57]. No ADAs developed, and mild injection-site reactions were the most common AE. However, non-cardiac chest pain associated with elevated c-reactive protein, alanine aminotransferase (ALT), aspartate aminotransferase and D-dimer but no thrombosis, was a severe AE in one patient, possibly attributed to fitusiran, resulting in discontinuation of the patient from the study [56, 57].

A phase I study evaluating the use of fitusiran in patients with inhibitors [59] demonstrated results similar to the previous phase I study. There were also mild, transient increases in the levels of D-dimer, ALT, aspartate aminotransferase and liver enzymes, these occurred more often with the higher 80 mg treatment dose. Patients with an ALT increase also had concurrent HCV infection. It was also recognised a reduced dose of aPCC was required to manage breakthrough bleeds with concurrent fitusiran use. Furthermore, an improvement in patient QoL was reported through use of the Haemophilia Quality of Life Questionnaire for Adults.

A phase II study[58] in HA and HB patients with and without inhibitors identified an ~ 80% decrease in antithrombin levels, rise in thrombin and reduction in ABRs, with 48% experiencing no bleeds. Injection-site reactions and rise in ALT were reported, however, possible confounders include fitusiran’s targeting of hepatocytes together with all affected patients being positive for Hepatitis C. During the extension, a death from cerebral venous sinus thrombosis occurred in one HA patient concomitantly given FVIII concentrate. The trial was temporarily halted until a risk mitigation protocol was constructed to reduce the occurrence of bleeding, thrombosis, and liver disease; and identify corresponding management. A study evaluating BPAs in fitusiran-treated plasma highlighted low doses were sufficient for haemostasis in breakthrough bleeds [60].

Perioperative implications of fitusiran were evaluated in four patients that had transitioned to the phase II study with antithrombin levels < 20%. It revealed minimal blood loss, successful haemostatic management in all cases and no thromboprophylaxis was used. Despite the small sample size, it was suggested that BPAs or factor dosing can be reduced for surgeries in patients on fitusiran [61].

Phase III (ATLAS) trials are currently ongoing, evaluating the efficacy of fitusiran in patients with HA and HB, with and without inhibitors [56, 58, 62]. Outcome parameters are ABRs, annualized spontaneous bleeds, annualized joint bleeds and QoL assessment.

Durability of haemostasis, suitability in both HA and HB regardless of inhibitor presence, reversibility with antithrombin, limited AEs, and no ADA formation, makes fitusiran a promising therapy. The once monthly subcutaneous injections and conversion to a milder bleeding phenotype would improve patient QoL and likely compliance compared to current regimes. Optimisation of dosing regimens, notably in breakthrough bleeds and emergency complications, still require further investigation.

## Concizumab

Concizumab (mAb 2021) is an IgG4 monoclonal antibody targeting tissue factor pathway inhibitor (TFPI) [63, 64]. It presents an alternative therapy for HA and HB patients, both with and without inhibitors [65].

**Fig. 2:**
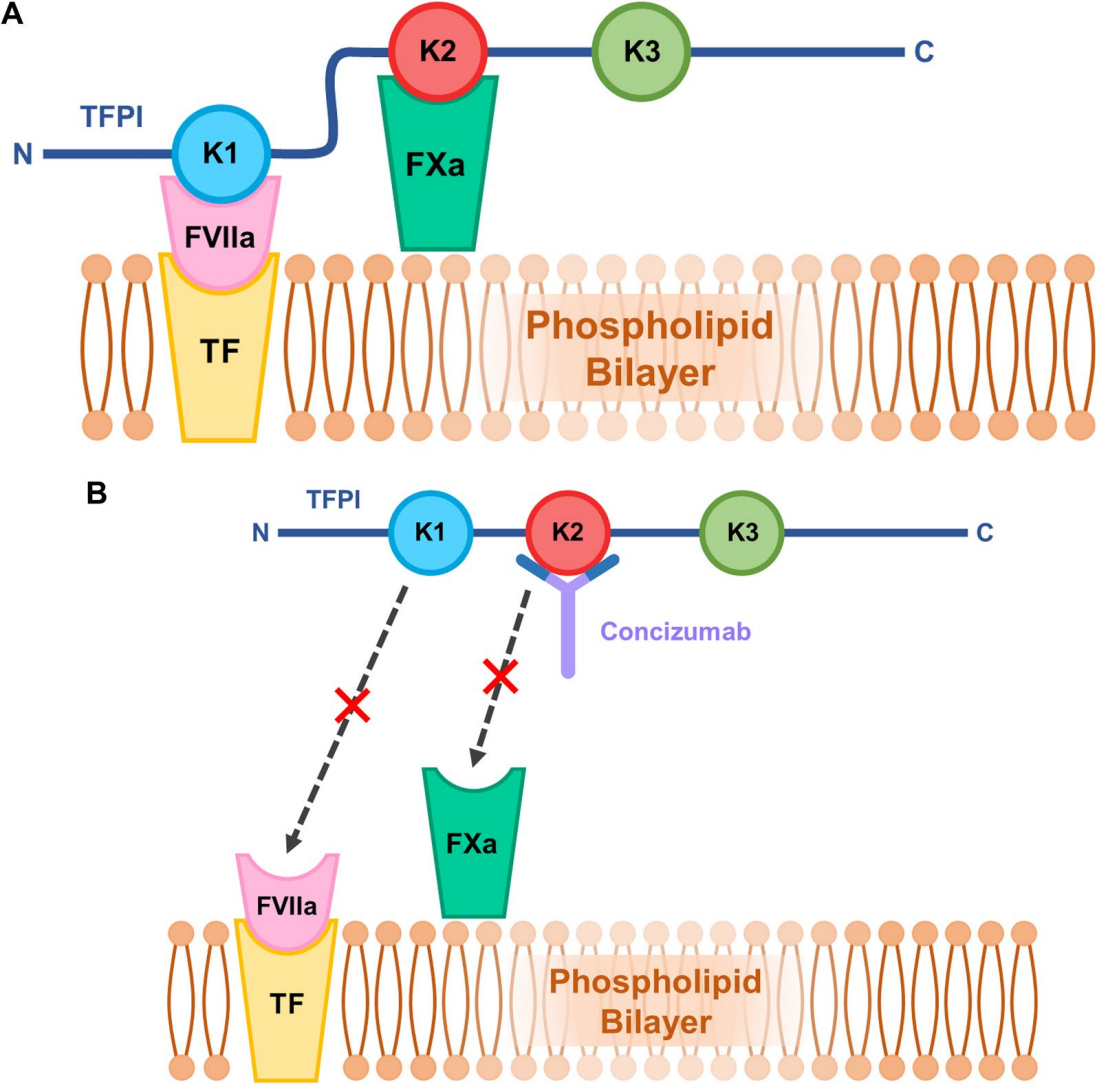
Mechanism of action of Concizumab relating to the different domains of TFPI. **A** physiological inhibition of the TF-FVIIa complex and the FXa complex through the binding of the K1 and K2 domains of TFPI to the respective coagulation factors. **B** concizumab binding to the K2 domain of TFPI and preventing the binding and inhibition of the TF-FVIIa complex and FXa by TFPI domains. *TFPI* tissue factor pathway inhibitor; *K1* Kunitz domain 1; *K2* Kunitz domain 2; *K3* Kunitz domain 3; *FVIIa* activated factor VII; *FXa* activated factor X; *TF* tissue factor

TFPI is a coagulation inhibitor comprising three Kunitz-type domains [66, 67]. It limits coagulation during the initiation of the coagulation cascade through inhibition of the tissue factor-activated factor VII (TF-FVIIa) complex, mediated by its first Kunitz domain (K1), and through FXa inhibition, mediated by its second Kunitz domain (K2) [68] (Fig. 2A).

Concizumab binds to an epitope on the K2 domain and subsequently prevents TFPI-mediated FXa inhibition [69] (Fig. 2B). By directly preventing FXa inhibition, concizumab permits the formation of greater quantities of thrombin in the initiation phase of coagulation [70–72]. Thrombin helps propagate subsequent activities of the coagulation cascade, and promotes fibrin production to stabilise a clot [70]. Additionally, TFPI-mediated TF-FVIIa complex inhibition is dependent on its inhibition of FXa [73, 74]. Thus, concizumab not only blocks FXa inhibition, but also indirectly prevents the inhibition of TF-FVIIa complex [69]. This is relevant since, physiologically, TF-FVIIa promotes FX activation, which consequently promotes further thrombin generation [72, 73].

Concizumab exhibits high affinity binding to the K2 domain, at a dissociation constant of 25 pM [63, 64]. It demonstrates a non-linear pharmacokinetic profile described as target-mediated drug disposition, whereby increasing doses of concizumab reduce its clearance rate since a smaller proportion of the drug is target-bound at higher concentrations [63, 64, 69, 71].

Preclinically [64, 75], concizumab was found to neutralize TFPI inhibition of FXa and promote FXa activation via TF-FVIIa, resulting in enhanced thrombin generation in a concentration-dependent manner. A rabbit haemophilia model demonstrated concizumab reduced cuticle bleeding with effects comparable to higher doses of conventional rFVIIa [64].

Explorer™4 and 5 evaluated the efficacy, safety, and immunogenicity of concizumab in HA and HB patients with inhibitors and HA patients lacking inhibitors respectively [65]. Both studies identified good safety profiles with mostly mild AEs, unrelated to concizumab, no issues occurring from concomitant rFVIIa or FVIII use for breakthrough bleeds, and no thromboembolic events [65]. Although six patients across both trials developed ADAs, three of which were neutralizing. The neutralizing antibody tests were transient. Antibody development was not associated with a change in clinical status or observed efficacy [65]. The procoagulant efficacy of concizumab was demonstrated across all three trial groups through reduced bleeding episodes; lower free TFPI; and increases in D-dimer, prothrombin fragment (F_1+2_), and thrombin generation [65].

Explorer™4 reported a statistically significant reduction in ABRs with concizumab, at 4.5 compared to 20.4 in the rFVIIa arm, and similar proportions of spontaneous and traumatic bleeds. Explorer™5 identified an estimated ABR of 7.0 and lower proportions of spontaneous and traumatic bleeds [65]. A prospective study (Explorer™6) is ongoing; investigating bleed frequency in patients with severe HA or HB, with or without inhibitors, whilst taking usual treatment [76].

Therapeutic monitoring of concizumab remains elusive, potentially involving investigation of residual TFPI activity through a prothrombin time or tissue factor-dependent assay [77]. Additionally, the perioperative implications from concomitant concizumab use are unknown.

The improvement to QoL from concizumab compared to conventional rFVIIa therapy was explored in a survey involving Explorer™4 participants taken at baseline and at 24 weeks [78]. The concizumab group experienced an improvement from baseline in the pain catastrophizing scale score and improvements in bodily pain, physical functioning, health perception, vitality score, and social functioning [78].

Other monoclonal antibodies, targeting TFPI, in development include marstacimab (previously PF-06741086) and MG1113. Marstacimab targets the K2 domain of TFPI. It has completed phase II clinical testing with encouraging results. MG1113, which, similarly to concizumab and marstacimab, targets the K2 domain, is currently undergoing phase I testing [79].

## Gene therapy

Gene therapy presents a novel and effective treatment modality for haemophilia, potentially bypassing complications of other therapies [80]. Gene therapy regimens consist of single infusions of a viral vector, which result in transduction of a gene coding for the deficient factor into patient hepatocytes [80, 81].

Current gene therapy regimens for haemophilia predominantly utilise adeno-associated virus (AAV) vectors to deliver the required gene [82]. Recombinant AAV (rAAV) consists of the AAV capsid surrounding a DNA sequence of intended incorporation, or transgene [81, 83]. rAAV-derived transgenes tend to be retained episomally, reducing risk of insertional mutagenesis [80]. The viral vector is non-pathogenic and unable to replicate without a helper virus, suggesting a safe method of transgene delivery [80], though AAV-mediated genotoxicity remains a debated issue. Potential increased risks associated with hepatocellular carcinoma development have been attributed to AAV-vector insertion in certain studies into murine models [80, 84–86]. More extensive long-term follow-up into human gene therapy studies must be conducted before the risk of AAV-mediated genotoxicity can be fully discerned [80, 85].

Single-stranded AAV (ssAAV) vectors incorporate a transgene by introducing a single-stranded DNA sequence into a nucleus, which uses host cell machinery to produce a complementary strand, resulting in formation of the functional gene [83, 87]. The more efficient self-complementary AAV (scAAV) vector bypasses the rate-limiting complementary strand formation; it carries a transgene consisting of two bound complementary single DNA strands, which anneal on nucleus entry to form a double-stranded gene [83, 87].

The FVIII gene requires shortening to allow successful packaging within an AAV vector [82]. To achieve this, the B-domain of the FVIII protein (which serves no function) is shortened to form an SQ-linker [81, 82, 88]. This allows for packaging within ssAAV; however, the transgene remains too large for delivery by scAAV, thus avoiding the theoretical increase in efficacy of this vector [81, 83]. This is contrary to the shorter FIX transgene, whose dedicated clinical trials commonly investigate delivery using scAAV, without need of truncation [81].

Various AAV serotypes have been investigated as vectors in clinical trials [82]. Use of the naturally occurring AAV8 serotype, in a vector carrying codon-optimised FIX via scAAV, has long-term efficacy in inducing FIX expression in HB patients [89]. This study demonstrated stable induction of FIX production in patients with severe haemophilia and an increase in FIX activity to 1–6 IU/dL for a median of 3.2 years [89]. HB patients showed drastic amelioration of the bleeding phenotype with an increase in FIX activity above 1 IU/dL [90]. A notable AE was an asymptomatic ALT increase amongst patients treated with a high-dose infusion (corrected with prednisolone), accompanied by a 50–70% loss in FIX expression [82, 89]. This suggested the occurrence of a cytotoxic cellular immune response targeting vector-infiltrated hepatocytes and influenced subsequent researchers to prescribe glucocorticoids, following gene therapy initiation or on ALT increase, to avoid similar complications [81].

Efficacy of AAV5 capsid use was demonstrated by a phase I/II trial [88], whereby a codon-optimised AA5 vector was used to integrate B-domain-deleted human FVIII of the SQ variant into severe HA patients. Two- and three-year follow-ups revealed a sustained increase in expression of FVIII in thirteen participants across two higher dose cohorts, and decreased mean ABRs [91]. The FVIII activity expressed after therapy was subject to annual decrease, raising concerns regarding the duration of effect [91].

The SPK-9001 vector utilises the bioengineered AAV-Spark100 capsid and a transgene encoding FIX-R338L (FIX-Padua) [92]. FIX-Padua results from a gain-of-function mutation which increases activity eightfold [93]. Results from a trial in ten patients with FIX activity of ≤ 2 IU/dL confirmed that using an FIX-Padua transgene would result in a similar increase in FIX activity at lower doses as compared to a normal variant [92]. Administering the vector at low dose is expected to minimise immune response without hindering efficacy [92].

Gene therapy was generally well-tolerated, although ALT increases were widely observed [88, 89, 91, 92], with occasional associated decreases in factor activity [89, 92]. These increases were transient following glucocorticoid treatment and were not consistently associated with an anti-capsid T cell response, raising inquiries as to the aetiology of the raised ALT [88, 89, 91, 92].

Despite the promising efficacy demonstrated in trials, limitations persist. Inefficacy of this treatment due to pre-existing anti-capsid neutralising antibodies has been reported [94]. This is worth consideration, given the potential ineffectiveness in a large proportion of the general population possessing these antibodies [92]. Due to the exclusion of patients with existing factor inhibitors in the outlined studies, treatment success in these patients is currently undetermined [81, 88, 89, 91, 92, 95]. However, it has been demonstrated in mouse subjects with existing FVIII inhibitors that gene therapy itself, in conjunction with rapamycin and anti-CD20, can be used to enhance immune tolerance induction [96].

Given the episomal retention of the rAAV transgene, hepatocyte turnover may result in decreased transgene-induced factor production over time, eventually necessitating AAV vector re-induction [83, 95]. This may complicate treatment outcomes, since the initial infusion of the AAV vector generally results in anti-capsid antibody production, rendering subsequent infusions with the same vector redundant or less effective[80, 88, 89, 91, 92]. Administration of synthetic vaccine particles encapsulating rapamycin in conjunction with AAV vector-mediated gene therapy has been shown in animal models to inhibit formation of anti-capsid antibodies, thus possibly potentiating subsequent infusions of the AAV vector if necessary [97].

This consideration highlights the requirement for long-term follow-up studies which are scarce, but promising; preliminary results of an 8-year follow-up showed maintained FIX expression [98]. Additionally, studies involving paediatric patients, whose hepatic growth and cell division indicates the likely need of repeated vector infusion [80–82]. Since the studies mentioned excluded participants under 18 years, an accurate conclusion regarding this matter cannot be made, presenting a vital consideration for the aims of future trials [88, 89, 91, 92]. In addition, as gene therapy becomes a more established therapy with potential use in clinical practice, concerns arise over the ability to produce enough AAV vector preparations should be considered. Hence development into the scalability of AAV vector production methods should be ongoing [80, 99].

The effects of increased production of coagulation factors, induced by AAV delivery of genetic material, on cellular stress and subsequent cellular toxicity is another consideration that must be made. However, studies have demonstrated in animal models that cellular stress was only observed following very high expression of the FVIII protein following AAV gene therapy, and limited evidence has been established to link this finding to increased levels of hepatocyte apoptosis [100, 101].

Gene editing strategies, whereby nucleases are used to remove the defective gene before viral vectors are utilised to insert a functional iteration of the removed gene, are currently being investigated [80, 102]. A clinical trial, involving the use of zinc-finger nucleases, to insert a functional FIX gene via an AAV6 vector to treat haemophilia B is awaiting publication of results [102, 103].

## Conclusion

Current factor replacement poses numerous issues, resulting in poor adherence and reduced QoL. Inhibitor development presents a key limitation to factor replacement. EHL products, emicizumab, fitusiran, and concizumab (summarised in Tables 1 and 2) appear effective in patients with and without inhibitors, and their longer half-lives enable less frequent injections. Furthermore, some of the therapies can be given subcutaneously, which is an added benefit. Although currently experimental, gene therapy has produced promising results, most notably the induction of long-lasting endogenous factor production.

**Table 2:** Summary and comparison of novel therapies discussed, outlining mechanism of action, route and frequency of administration, advantages, limitations, and the current licensing status

Therapy	Mechanism of action	Route of administration	Frequency of administration	Advantages	Limitations	Licensing status
Emicizumab	Bispecific antibody mimicking co-factor function of FVIII	Subcutaneous	Once a week, Twice a week, or Monthly	Can be used in patients with FVIII-inhibitors No need for peripheral venous access Reduced frequency of administration Reduced cost of treatment No need for routine laboratory monitoring, practical to use Good safety profile	Interferes with the assays used in laboratory monitoring Insufficient to treat large bleeds on its own, additional haemostatic measures required	Licensed for use in HA patients with and without inhibitors
Fitusiran	GalNAc-siRNA conjugate	Subcutaneous	Once monthly	Application in both HA and HB patients with and without inhibitors Reduced frequency of administration No need for peripheral venous access Good safety profile	More research required for dose selection and management of breakthrough bleeds Lack of paediatric trial data	In phase III of development
Concizumab	Anti-TFPI monoclonal antibody	Subcutaneous	Once daily	Application in both HA and HB patients with and without inhibitors No need for peripheral venous access Good safety profile Improvement in patient QoL	Daily administration Further research required into therapeutic monitoring Further research required into implications for surgery	In phase III of development
Gene therapy	Transduction of a gene coding for deficient factor into patient hepatocytes	Intravenous	Single dose	Application in both HA and HB patients Reduced frequency of administration Improvement in patient QoL Potential role for immune tolerance induction	Lack of long-term follow up Further investigations required into a wider range of patient demographics Potential for degradation by anti-capsid antibodies Diminishing efficacy over time	Undergoing clinical trials

*FVIII* factor VIII; *HA* haemophilia A; *GalNAc-siRNA* *N*-acetylgalactosamine-small interfering RNA; *HB* haemophilia B; *TFPI* tissue factor pathway inhibitor; *QoL* quality of life

Despite the high efficacy and promising outlook for the therapies outlined in this review, conventional factor replacement remains the most prevalent haemophilia treatment modality. In resource-constrained countries, access to replacement factors is still greatly limited [104], thus the introduction of new and sophisticated therapies with higher costs are likely to be inaccessible to a large proportion of haemophilia patients globally.

In conclusion, novel haemophilia therapies offer an arsenal of treatment options and could revolutionise the future of haemophilia management. However, the infrastructure facilitating worldwide availability of these novel treatments remains a barrier.

## Data Availability

Not applicable.
